# Adult Influence on Juvenile Phenotypes by Stage-Specific Pheromone Production

**DOI:** 10.1016/j.isci.2018.11.027

**Published:** 2018-11-20

**Authors:** Michael S. Werner, Marc H. Claaßen, Tess Renahan, Mohannad Dardiry, Ralf J. Sommer

**Affiliations:** 1 Department of Evolutionary Biology, Max Planck Institute for Developmental Biology, Tübingen 72076, Germany

**Keywords:** Physiology, Genetics, Cell Biology, Developmental Biology

## Abstract

Many animal and plant species respond to population density by phenotypic plasticity. To investigate if specific age classes and/or cross-generational signaling affect density-dependent plasticity, we developed a dye-based method to differentiate co-existing nematode populations. We applied this method to *Pristionchus pacificus*, which develops a predatory mouth form to exploit alternative resources and kill competitors in response to high population densities. Remarkably, adult, but not juvenile, crowding induces the predatory morph in other juveniles. High-performance liquid chromatography-mass spectrometry of secreted metabolites combined with genetic mutants traced this result to the production of stage-specific pheromones. In particular, the *P. pacificus*-specific di-ascaroside#1 that induces the predatory morph is induced in the last juvenile stage and young adults, even though mouth forms are no longer plastic in adults. Cross-generational signaling between adults and juveniles may serve as an indication of rapidly increasing population size, arguing that age classes are an important component of phenotypic plasticity.

## Highlights

• Novel vital dye method for tracking mixed nematode populations
• Adult, but not juvenile, crowding induces the omnivorous morph in *P. pacificus*
• Omnivorous morph-inducing pheromones are produced late in development
• Age class is an important component of density-dependent phenotypic plasticity

## Graphical Abstract

**Figure:**
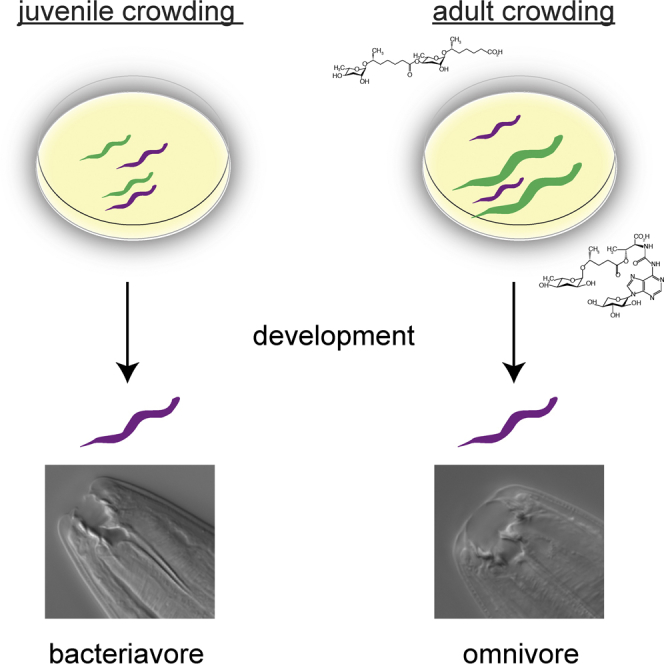

## Introduction

Population density is an important ecological parameter, with higher densities corresponding to increased competition for resources (Hastings, 2013). In addition to density-dependent selection (MacArthur, 1962, Travis et al., 2013), which operates on evolutionary timescales, some organisms can respond dynamically to population density through phenotypic plasticity. For example, plants can sense crowding by detecting the ratio of red (chlorophyll absorbing) to far red (non-absorbing) light, and respond by producing higher shoots (Dudley and Schmitt, 2015). Locusts undergo solitary to swarm (i.e., gregarious) transition as a result of increased physical contact (Pener and Simpson, 2009, Simpson et al., 2001, Slogget and Weisser, 2002). Intriguingly, population density can also have cross-generational effects, defined here as the density of one age group affecting the phenotypes of another. For example, adult crowding of the desert locust *Schistocerca gregaria* (Maeno and Tanaka, 2008, Simpson and Miller, 2007) and migratory locust *Locusta migratoria* (Chen et al., 2015, Ben Hamouda et al., 2011) also influences the egg size, number, and morphology of their progeny, high population densities of red squirrels elicit hormonal regulation in mothers to influence faster-developing offspring (Dantzer et al., 2013), and crowding in aphids can induce winged progeny from flightless parents (Slogget and Weisser, 2002, Sutherland, 1969). In many species, population density and cross-generational signaling are communicated by pheromones; however, the precise nature, mechanisms of induction, age specificity, and exact ecological role are not well understood.

**Figure 1:**
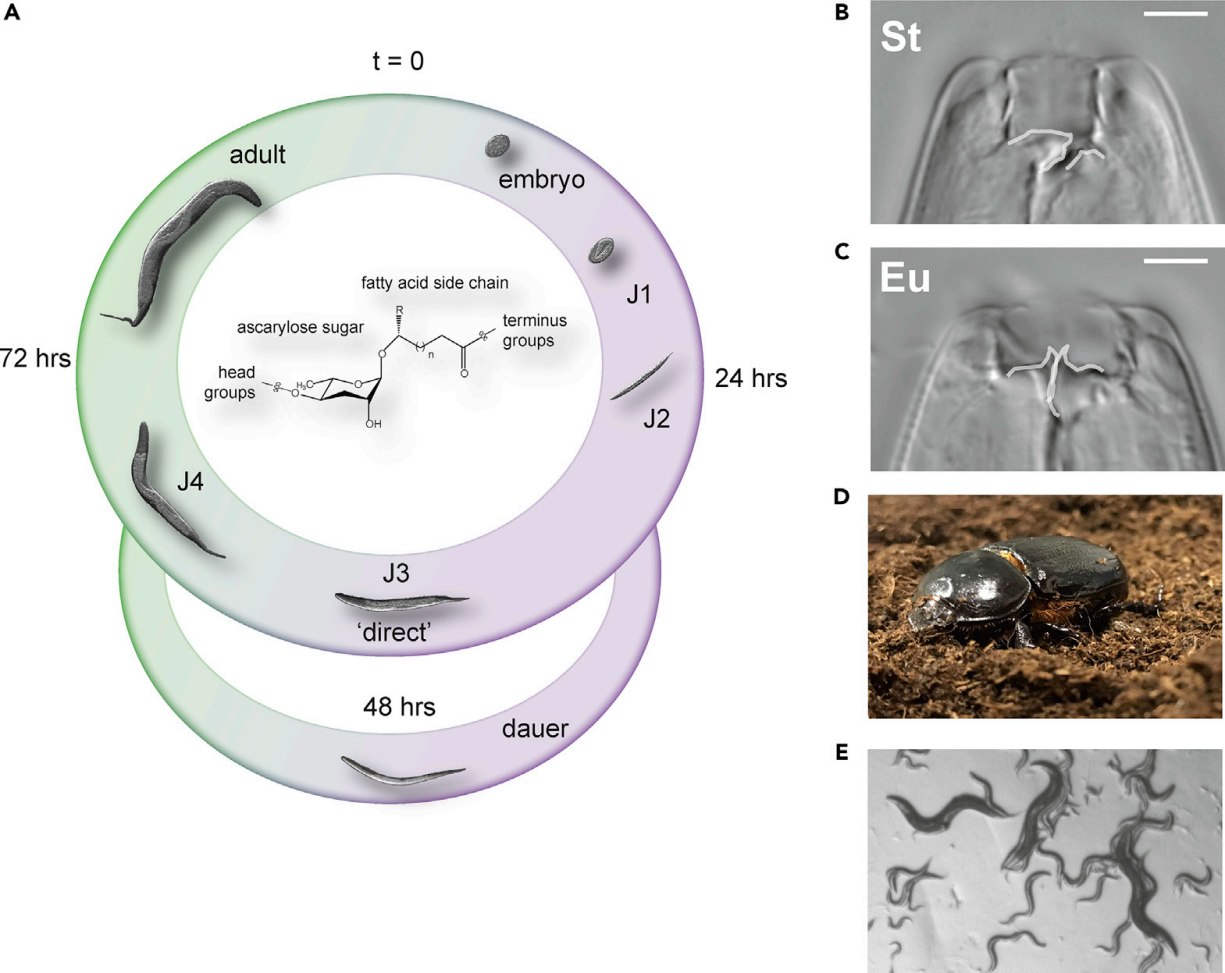
Life Cycle and Developmental Plasticity of the Model Nematode *Pristionchus pacificus* (A) The life cycle of *P. pacificus* consists of four juvenile stages (J1–J4) until sexual maturation (adult hermaphrodites). Like many nematodes *P. pacificus* can enter a long-living “dormant” dauer state that is resistant to harsh environmental conditions. The decision to continue through the direct life cycle or enter dauer is regulated by small-molecule-excreted ascarosides (chemical structure adapted from Butcher, 2017). (B and C) (B) *P. pacificus* can also adopt one of two possible feeding structures; either a microbivorous narrow mouth (stenostomatous, St) or (C) an omnivorous wide mouth (eurystomatous, Eu) with an extra tooth that can be utilized to kill and eat other nematodes or fungi. White lines indicate the presence of an extra tooth (right side) in the Eu morph or its absence in the St morph, and the dorsal tooth (left side), which is narrow and elongated (flint-like) in St and hook like in Eu. Scale bar, 5 μM. (D) *P. pacificus* is often found in a necromenic association with beetles (e.g., shown here *Oryctes borbonicus,* photo taken by Tess Renahan) in the dauer state and resumes the free-living life cycle upon beetle death to feed on the ensuing microbial bloom. (E) RSC017 mixed-staged worms on agar plates.

Nematodes are a powerful model system to investigate the mechanisms of density-dependent plasticity because many small molecule pheromones that affect plastic phenotypes have been characterized (Butcher, 2017, Butcher et al., 2007, von Reuss et al., 2012). For example, in the model organism *Caenorhabditis elegans,* high population densities induce entry into a stress-resistant dormant “dauer” stage (Fielenbach and Antebi, 2008). The decision to enter dauer was revealed to be regulated by a family of small molecule nematode-derived modular metabolites (NDMMs) called ascarosides that act as pheromones (Butcher et al., 2007, Butcher et al., 2008, Jeong et al., 2005). Ascarosides consist of an ascarylose sugar with a fatty acid side chain and modular head and terminal groups (Figure 1A). The level and composition of ascarosides were later shown to be dependent on sex (Chasnov et al., 2007, Izrayelit et al., 2012) and development (Kaplan et al., 2011), although it is thought that early larval development into dauer can be induced by pheromones from all developmental stages (Golden and Riddle, 1982). Subsequent studies revealed that specific NDMMs also regulate other life history traits, such as mating (Chasnov et al., 2007, Izrayelit et al., 2012), social behavior (Srinivasan et al., 2012), and developmental speed (Ludewig et al., 2017). Although NDMMs are broadly conserved (Choe et al., 2012, Dong et al., 2018, Markov et al., 2016), inter- and intraspecific competition have driven the evolution of distinct response regimes (different levels of sensitivity to the same pheromone, or sensitivity to different pheromones) for the same phenotypes (Bose et al., 2014, Choe et al., 2012, Diaz et al., 2014, Falcke et al., 2018, Greene et al., 2016). In addition, distinct plastic phenotypes have evolved that are regulated by more complex ascaroside structures (Bose et al., 2012).

In *Pristionchus pacificus,* a soil-associated nematode that is reliably found on scarab beetles (Figure 1A) (Herrmann et al., 2006, Herrmann et al., 2007, Sommer and McGaughran, 2013), an ascaroside dimer (dasc#1) that is not found in *C. elegans* regulates the development of a predatory mouth form (Bento et al., 2010, Bose et al., 2012, Sommer et al., 2017). Mouth-form plasticity represents an example of a morphological novelty that results in predatory behavior to exploit additional resources and kill competitors. Specifically, adult *P. pacificus* exhibit either a narrow stenostomatous (St) mouth (Figure 1B), which is restricted to bacterial feeding, or a wide eurystomatous (Eu) mouth with an extra denticle (Figure 1C), which allows for feeding on bacteria and fungi (Sanghvi et al., 2016), and predation on other nematodes (Wilecki et al., 2015). This type of phenotypic plasticity is distinct between direct, non-arrested development and indirect (dauer) development because the mouth form decision results in two alternative life history strategies in the adult (for review, see Sommer & Mayer, 2015). Recent studies in *P. pacificus* have begun to investigate the dynamics and succession of nematodes on decomposing beetle carcasses to better understand the ecological significance of mouth-form plasticity (Meyer et al., 2017). These studies revealed that on a carcass (Figure 1D), *P. pacificus* exits the dauer diapause to feed on microbes, and then re-enters dauer after food sources have been exhausted, displaying a “boom-and-bust” ecology (Meyer et al., 2017, Sommer and McGaughran, 2013). Presumably different stages of this succession comprise different ratios of juveniles and adults, and recognizing the age structure of a population as a juvenile could provide predictive value for adulthood. However, it is unknown whether the mouth-form decision is sensitive to crowding by different age classes (example of crowding by different age groups, Figure 1E). More broadly, whereas age classes are known to be important for population growth and density-dependent selection (Hastings, 2013, Charlesworth, 1994, Charlesworth, 1972), their role in phenotypic plasticity has thus far been largely unexplored.

Although nematodes have many experimental advantages, including easy laboratory culture and advanced genetic, genomic, and the aforementioned chemical tools, their small size has made investigations at the organismal level and in experimental ecology challenging. For example, no *in vivo* methodologies are currently available to label distinct populations without the need for transgenics, which is only available in select model organisms such as *C. elegans*, *P. pacificus*, and some of their relatives. Here, we combine a novel dye-staining method with the first developmental pheromone profiling in *P. pacificus* to study the potential effects of age on density-dependent plasticity. This vital dye method allows tracking adults with juveniles, or juveniles with juveniles, and can be applied to any nematode system that can be cultured under laboratory conditions. In contrast to dauer, we found that mouth form is strongly affected by cross-generational signaling. Specifically, only adult crowding induces the predatory morph, which is controlled by stage-specific pheromones.

## Results

### A Vital Dye Method for Labeling Nematode Populations

**Figure 2:**
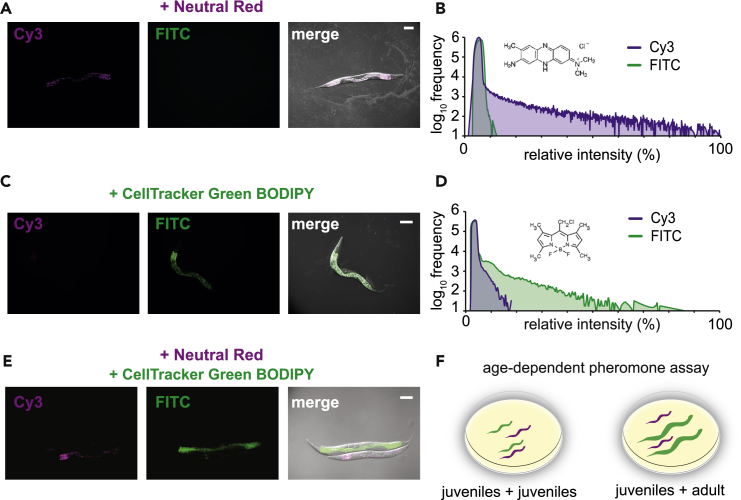
Vital Dye Method in Nematodes Allows Mixing Different Populations Together (A) Neutral Red-stained adults (0.005% for 3 hours) imaged with Cy3 and FITC excitations and filters, and merged with DIC. (B) An example of the relative intensities of fluorescence displayed as a histogram with the chemical structure of Neutral Red. (C) CellTracker Green BODIPY (Thermo)-stained adults (50 µM for 3 hours) imaged with Cy3 and FITC excitations and filters, and merged with DIC. (D) An example of the relative intensities of fluorescence displayed as a histogram with the chemical structure of CellTracker Green BODIPY. (E) Combined worms from Neutral Red and CellTracker Green BODIPY staining on the same slide, merged with DIC. (F) Age-dependent functional pheromone assay: experimental juveniles were stained with neutral red and challenged with CellTracker Green BODIPY-stained juveniles or adults on standard condition Nematode Growth Media (NGM) agar plates seeded with 300 μL OP50 *E. coli*. Three days later, only red-positive and green-negative adults were phenotyped.

To directly test if different age groups of *P. pacificus* influence mouth form, we required two synchronized populations to co-habit the same space, yet still be able to identify worms from different age groups. To do so, we developed a dye-staining methodology to robustly differentiate between nematode populations. After trying several vital dyes, we identified that neutral red (Thomas and Lana, 2008) and CellTracker Green BODIPY (Thermo) stain nematode intestines brightly and specifically to their respective channels (Figures 2A–2E and S1, Transparent Methods). These dyes stained all nematodes tested including *C. elegans* (Figure S2) and dauer larvae (Figures S3A and S3B). Both dyes lasted more than 3 days and neutral red >5 days (Figures S3C–S3G), allowing long-term tracking of mixed nematode populations. Importantly, neither neutral red nor CellTracker Green staining affected viability, developmental rate, or the formation of specific morphological structures, such as *P. pacificus* mouth form (Figure S4). Thus, neutral red and CellTracker Green allow specific labeling of worm populations to study age-dependent effects on phenotypes.

### Adult but Not Juvenile Crowding Induces the Predatory Mouth Form in *P. pacificus*

**Figure 3:**
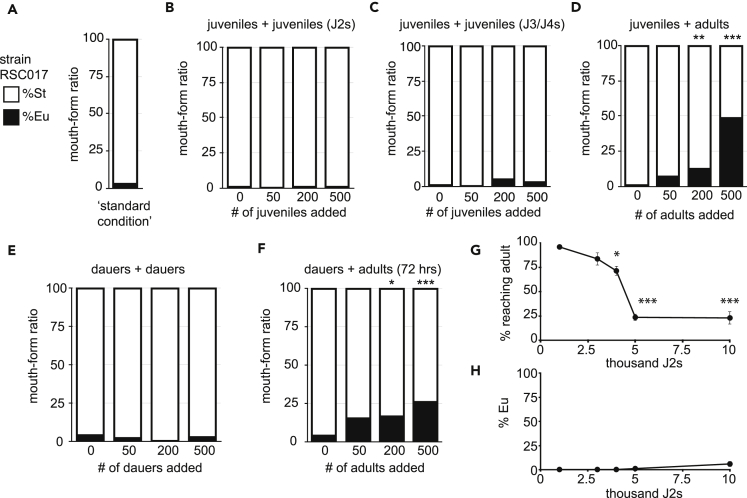
Vital Dye Method Demonstrates Adult-Specific Density Effect on Mouth Form (A–F) (A) The wild isolate RSC017 grown in standard conditions (5 young adults passed to fresh plates, progeny phenotyped 4 days later) are highly stenostomatous (<10%, *n* = 102). Mouth form ratios of neutral red-stained J2s (B–D) and dauers (E and F), with increasing number of CellTracker Green-stained competitors (total number of animals *n* > 100 per experiment, with 3–5 independent biological replicates for J2 and adult crowding, and 2 for J3/J4s). Overall significance between strain and age was determined by a binomial linear regression (see Transparent Methods), and pairwise comparisons were assessed by Fisher's exact test on summed Eu counts (^∗∗∗^p < 0.001, **p < 0.01, *p < 0.05). Mouth forms were phenotyped at 40–100× on a Zeiss Axio Imager 2 light microscope. (G and H) (G) Percent reaching adulthood and percent Eu of those that reached adulthood (H) after increasing numbers of J2s were added to standard 6-cm Nematode Growth Media (NGM) agar plates with 300 μL OP50 *E. coli* bacteria (n = 2 biological replicates, with total *n* > 200 for percent reaching adulthood, and total n > 100 for mouth form. Significance was determined by a binomial regression; Error bars represent standard deviation of the two biological replicates).

To assess potential intra- or inter-generational influence on *P. pacificus* mouth form, we stained 200 juveniles stage 2 (J2s) of the highly St strain RSC017 (Figure 3A) with neutral red and added an increasing number of CellTracker Green-stained RSC017 adults or juveniles (J2s or J3/4s) (Figure 2F). After 3 days, we phenotyped red animals that had developed into adults. Almost half (48%) of the population developed an Eu mouth form with 500 adult animals, compared with less than 4% with 500 J2 or J3/4 juveniles (*n* > 100 from 2–5 independent biological replicates; for display, summed percentages are shown in Figures 3B–3D). We performed a direct statistical comparison between crowded plates and controls (no added crowding animals) for every number and stage of crowding. After multiple testing corrections, only 200 and 500 adult-crowded plates yielded significant differences compared with control (un-crowded) plates (*Bonferroni-corrected* p = 6.9 × 10^−3^ and <2.2 × 10^−16^, respectively, Fisher's exact test on Eu counts). To ascertain if there is a general difference between juvenile or adult crowding, we performed a binomial regression on replicate Eu count data, with stage (J2–J4 versus adults) and number of crowding animals included as fixed effects (Transparent Methods, Table S1). Indeed, we observed a significant difference between adult and juvenile crowding and the incidence of Eu morphs (p = 1.32 × 10^−2^).

We were also curious if dauers, which have a thickened cuticle and represent a distinct stage in the boom-and-bust life cycle of nematodes, could still respond to adults. Indeed, the same trend that was observed with juveniles was seen with dauers; only 200 and 500 adults significantly induced the Eu mouth form, albeit to a more muted extent (Figures 3E and 3F) (*Bonferroni-corrected* p = 2.4 × 10^−2^ and 7.3 × 10^−5^, respectively; Fisher's exact test; and binomial regression between dauer and adult crowding p = 2.96 × 10^−3^). With a total of 200 dauers and 500 adults, 25.7% of dauers became Eu, whereas only 1.8% of dauers become Eu on a plate containing 700 dauers (and no adults) (Figure 3F). Collectively, these data indicate that adult crowding specifically induces the Eu mouth form. However, it should be noted that because of the difficulty in obtaining a pure J4 culture from RSC017s, we cannot rule out that crowding by large numbers of J4s could also induce the Eu morph.

Even though we did not detect a mouth-form switch in large populations of J2s or dauers, and food was still visible on plates containing the most animals (500 “crowders”), we could not completely rule out the possible effect of food availability on mouth form. As a proxy for starvation, we conducted assays with greatly increased numbers of juveniles from 1,000 to 10,000 that would rapidly deplete bacterial food. We noticed a stark cliff in the fraction of animals that reach adulthood at 4,000–5,000 juveniles, arguing that food is a limiting resource at this population density (Figure 3G). Importantly, however, in these plates we still did not see a shift in mouth form (Figure 3H) (p = 0.99, binomial regression, Table S1). With an overwhelming 10,000 worms on a plate, 5.8% were Eu, compared with 48% in the presence of only 500 adults. Although longer-term starvation may have an impact on mouth form, under our experimental conditions it appears to be negligible.

### Late-Stage Secretions Induce the Eu Mouth Form

**Figure 4:**
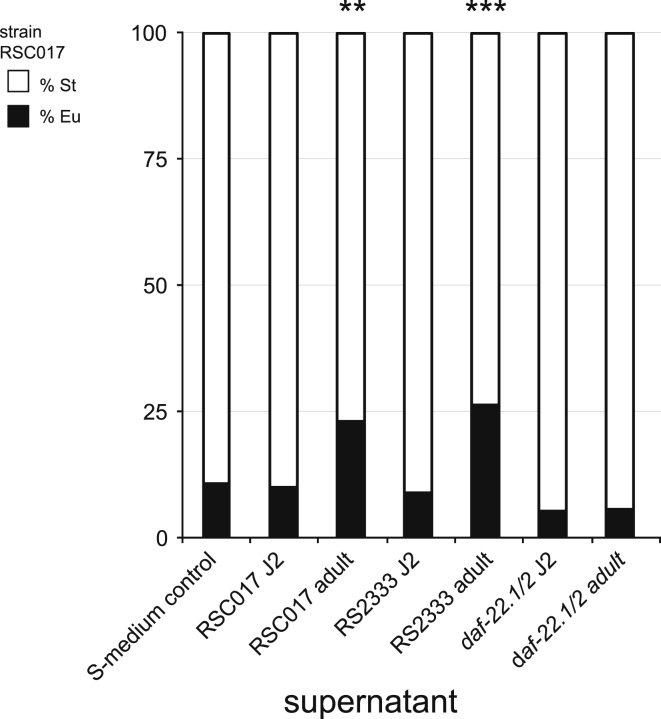
Late-Stage Secretions Induce Predatory Morph in Juveniles Highly St strain RSC017 juveniles were exposed to 24- and 72-hr supernatants of its own strain, and to the 24- and 72-hr supernatants of the highly Eu strain RS2333. Mouth form was phenotyped 3 days later. Worms exposed to 24-hr secretions remained highly St, whereas worms exposed to 72-hr secretions had a small but significant increase in Eu morphs (p < 0.05, Fisher's exact test). Supernatants from the double mutant *daf-22.1/2*, which has deficient ascaroside pheromone production (Golden and Riddle, 1985, Markov et al., 2016), did not elicit increases in Eu from either 24- or 72-hr supernatants. Worms exposed to the S-media control also remained highly St. n = 4 independent biological replicates for RS2333 and *daf-22.1/2* secretions, and n = 2 independent biological replicates for RSC017 secretions, with an average count of 55 animals per replicate. For display, total Eu and St counts are presented as percentages (*** p < 0.001, **p < 0.01).

As the mouth-form decision in *P. pacificus* can be influenced by NDMMs (Bose et al., 2012), we wondered if the difference in Eu induction between adults and juveniles resulted from differences in secreted pheromones. To test this hypothesis, we added secretions from 24- and 72-hr cultures of RSC017 and the laboratory strain RS2333 (which is highly Eu) to RSC017 juveniles. We found that the 72-hr (late juvenile stage 4/adult, Figure S4H; Werner et al., 2017) secretions from both strains led to a significant increase in the Eu morph relative to the 24-hr (early juvenile J2) secretions (p = 5.27 × 10^−6^, 1.33x10^−3^, respectively, Fisher's exact test on Eu counts relative to S-medium controls, n = 2-4 biological replicates; for display, summed percentages are shown in Figure 4). To confirm that the effect was caused by ascaroside pheromones, we exposed RSC017 juveniles to supernatant from a *P. pacificus daf-22.1;daf-22.2* double mutant, which exhibits virtually no ascaroside production in both *C. elegans* and *P. pacificus* (Golden and Riddle, 1985, Markov et al., 2016). Again, early juvenile secretion had no impact on Eu frequency, but in contrast to wild-type supernatants, we observed no significant increase in Eu frequency with the 72-hr secretions (p = 0.8324, Fisher's exact test, Figure 4). Thus, late-stage NDMMs induce development of the Eu mouth form.

### Developmental-Staged NDMM Profiles Reveal Age-Specific Synthesis of dasc#1

**Figure 5:**
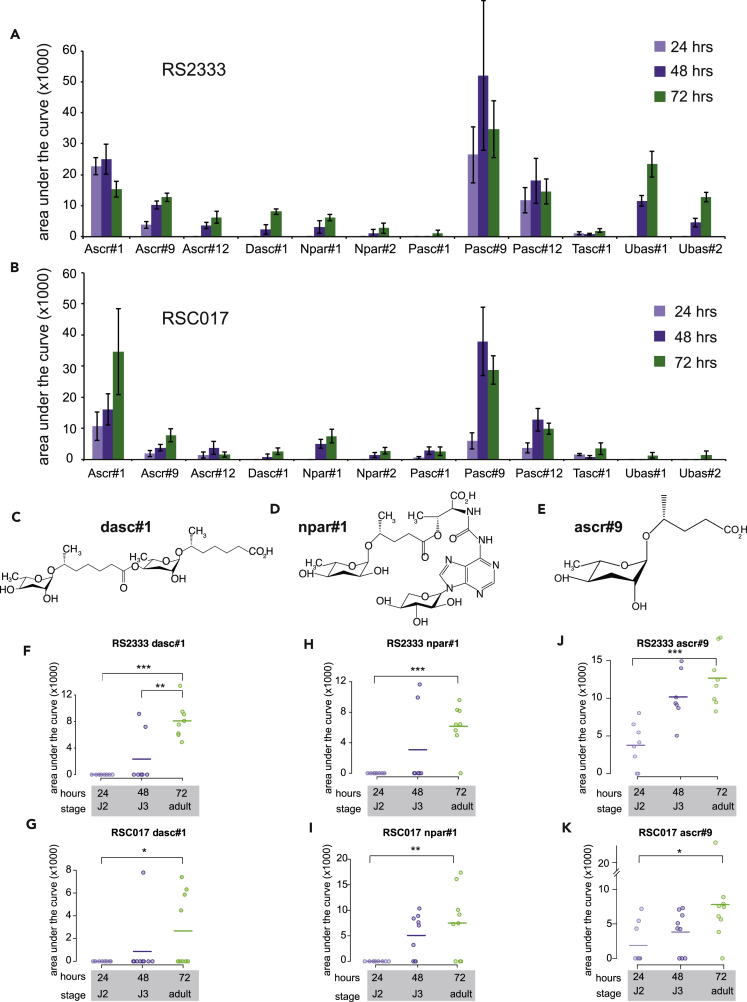
Time-Resolved Nematode-Derived Modular Metabolites (NDMMs) in *Pristionchus pacificus* (A and B) (A) Time-resolved secretion profile of nematode-derived modular metabolites from the wild-type laboratory strain RS2333 and (B) wild isolate RSC017. In both strains, at 24 hr cultures represent predominantly J2 stage worms, at 48 hr a mix of J2–J4, and at 72 hr predominantly adults in RSC017 (90%, Figure S4H) and a mix of J4/adults in RS2333 (Werner et al., 2017). Data are presented as the mean of 8 (RS2333) and 9 (RSC017) biological replicates, and error bars represent the standard error of mean (SEM). (C–E) Chemical structures of age-specific NDMMs (C) dasc#1, (D) npar#1, and (E) ascr#9, as described in the Small Molecule Identifier Database (http://www.smid-db.org/), produced in ChemDraw. (F–K) Time-resolved abundance of (F and G) dasc#1, (H and I) npar#1, and (J and K) ascr#9 NDMMs in RS2333 and RSC017. Each data point represents a biological replicate, and for comparison with (A and B) lines represent mean abundance. p values calculated by a 2-tailed Student's t test (***p < 0.001, **p <0.01, *p <0.05).

Next, we investigated whether the different effects of early and late pheromones are ones of dosage, or of identity. To determine the potential age-specific differences in pheromones, we profiled *P. pacificus* NDMM levels in two strains and at three time points throughout development with high-performance liquid chromatography-mass spectrometry (Figures 5A, 5B, and S5). We performed a linear regression with the area under the curve of each NDMM chromatogram (Figure S5A) as the response variable. Stage and strain were modeled as fixed effects, and because we performed separate regression analyses for each pheromone, we adjusted the resulting p values for multiple testing using false discovery rate (FDR) (see Table S2 for p and FDR values between stage and strain). We observed that among developmental stages there were significant differences in the levels of ascr#9, ascr#12, npar#1, and dasc#1, and that dasc#1, ubas#1, and ubas#2 are affected by both stage and strain (FDR<0.05). Interestingly, dasc#1 is the most potent known Eu-inducing pheromone when tested as a single synthesized compound, whereas npar#1 is both an Eu- and a dauer-inducing pheromone (Bose et al., 2012). Closer inspection revealed dasc#1, npar#1, and ascr#9 increase throughout development in both strains, and dasc#1 peaks at 72 hr in RS2333 (Figures 5C and 5D and 5F–5I, p < 0.05, Student's two-tailed t test between 72 and 24 hr for each NDMM in both strains, and 72 and 48 hr for dasc#1 in RS2333, Table S3). Intriguingly, the trajectory of dasc#1 appeared “binary/off-on” in both strains; in some replicates dasc#1 levels were undetectable, whereas others were high and virtually no replicates exhibited intermediate levels (Figures 5F and 5G). In fact, our statistical model for dasc#1 fits better if we assume cubic rather than linear growth (model difference Akaike information criterion, *ΔAIC* = 3.958). In contrast, ascr#9, which was also statistically increased but does not affect known plastic phenotypes (Bose et al., 2012), displayed a more gradual increase in both strains (Figures 5E, 5J, and 5K), and the model fits better with linear growth (AIC_linear_ – AIC_cubic_ = −1.208). Meanwhile, the induction pattern of npar#1 appears particular to each strain, although our linear model did not detect significant strain effects. Thus the kinetics of induction appears to be NDMM specific, which may be related to their roles in phenotypic plasticity.

We were interested to know if there was a transcriptional signal that would correlate with the increase in NDMMs throughout development. An analysis of previously published RNA sequencing data (Baskaran et al., 2015) reveals an ∼5-fold increase in transcription of the thiolase *Ppa-daf-22.1* (Figure S6A) between J2 and J4/adults, the most downstream enzyme in the β-oxidation pathway of ascaroside synthesis. However, this enzyme is responsible for the last step in synthesizing many NDMMs in addition to dasc#1, npar#1, and ascr#9, so other enzymes must also be involved, and identifying them is an area of active research.

In principle, the increase in abundance of dasc#1, npar#1, and ascr#9 throughout development could be a result of a concomitant increase in body mass. We used WormSizer (Moore et al., 2013) to measure the size of RSC017 animals from each time point and then normalized NDMM abundances by volume. We found a 1.1-fold difference in body volume between 24- and 48-hr samples, and a 1.3-fold difference between 24- and 72-hr samples. However, normalizing by these factors did not affect the significance of dasc#1, npar#1, or ascr#9 between time points (Tables S3 and S4; Figures S6B–S6D). We also suspect that size is not the only factor because no other compounds significantly increased throughout development in our linear model. Finally, we profiled the endo-metabolome of eggs and found appreciable amounts of ascr#1, #9, and #12 and pasc#9, but little to no traces of other ascaroside derivatives (Figure S5C), suggesting age-specific synthesis, rather than release from ascarosides already present in eggs/J1. Together, these results suggest that the observed increase in ascr#9, npar#1, and dasc#1 over time corresponds to age-specific production. The observation that dasc#1 is produced specifically during the juvenile-to-adult transition is especially intriguing because adults are no longer able to switch mouth forms, hinting at cross-generational signaling.

## Discussion

Here, we introduce a novel dye-based method that allowed us to assess cross-generational influence on mouth form. Our results demonstrate that adult crowding induces the Eu predatory morph, and that this effect is, at least partially, a result of age-specific pheromones. In doing so, we provide the first multi-stage time series of pheromone production in *P. pacificus*, which shows that dasc#1 exhibits a surprising switch-like induction pattern. Collectively, our results suggest that adults represent a “critical age group” with respect to phenotypic plasticity. The fact that adults also represent the critical age group with respect to population density (Charlesworth, 1972) may explain their outsized contribution to induction of the Eu morph. The presence of adults may indicate rapidly decreasing bacterial resources, and thus developing the Eu morph will allow worms to exploit additional resources and kill competitors.

Our developmental profiling revealed an increase in two NDMMs that affect plastic phenotypes. Given that J4s can produce dasc#1/npar#1, we believe the lack of effect of the J3/J4 stage compared with adults in our mixed-culture assay simply reflects the more consistently present and higher amounts of dasc#1/npar#1 produced at 72 hr and experienced for longer periods of time. The observation that this trend occurs regardless of body size implies that these molecules are programmed for stage-specific production. The “off-on” induction kinetics might reflect a population-level feedback loop, wherein the production of excess pheromones is based on a threshold level of previously produced pheromones. The variability observed at 48 hr for dasc#1/npar#1 might reflect biological variability in developmental timing and/or technical variation in staging. It is also worth noting that although npar#1 is the major dauer-inducing pheromone in *P. pacificus* (Bose et al., 2012), we did not observe dauer juveniles in any of our dye-crowding assays. Thus, it seems that mouth-form phenotype is the first-level plastic response to population density. Presumably higher concentrations are required for dauer induction, reflecting a calculated response strategy depending on the level of crowding or duration of starvation. Interestingly, the effect of 72-hr supernatants was noticeably less (23%–26% Eu) than the physical presence of adult worms (up to 48% with only 500 adults). It is difficult to compare pheromone concentrations between experiments, but presumably worms in the vital dye assay experienced a greater local concentration as they were in direct contact with each other for longer periods of time, and were also older than the 72-hr supernatant assayed in our pheromone profiling. However, it is also formally possible that other factors, like increased physical contact, can induce the Eu morph.

The maximum levels of Eu reached in our mixed culture experiment was ∼50% with 500 adults, begging the question if this could be pushed further by using greater levels of crowding. However, this proved technically difficult due to food constraints with excess worms. Adding more food (OP50 LB) began to decrease the integrity of the agar, which made recovering animals for phenotyping difficult. Importantly, adults do not seem capable of eating other adults, which might otherwise push the Eu frequency even higher as a defense strategy. We also suspect that there are unknown trade-offs between the Eu and St mouth forms, which may manifest in a “ceiling” of the Eu frequency even under more crowded conditions.

Among the many environmental influences on mouth form (Werner et al., 2017), population density and starvation are perhaps the most ecologically relevant. However, teasing apart these two factors has been a challenge (Bento et al., 2010). Here, we demonstrate that whereas a strong shift is observed with age-specific pheromones, no such effect was seen under limited resource conditions. Thus, age-specific crowding is sufficient to induce the Eu mouth form. Nevertheless, this does not preclude that long-term starvation could also have an effect. Determining the relative contributions of these factors to mouth form will be important to better understand the sophisticated ecological response strategies of *P. pacificus*, nematodes, and phenotypic plasticity in general.

**Figure 6:**
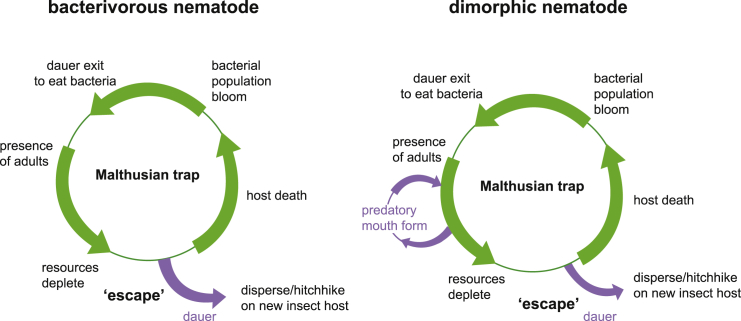
Conceptual Model of the Role of Critical Age Classes in Mouth-Form Phenotypic Plasticity Conceptual life cycle models of monomorphic or dimorphic mouth-form nematodes. In an isolated niche such as a decaying insect carcass, at some point microbial food supplies will run out, leading to a Malthusian catastrophe. Nematodes escape this trap by entering the dauer state and dispersing, and re-starting the cycle. Dimorphic nematodes may sense the impending “catastrophe” earlier by recognizing an abundance of adults in the population, and switching to the Eu morph to exploit new resources and kill competitors. By analogy to economic models, the mouth-form switch is a technological innovation to temporarily escape a Malthusian resource trap.

Why do adults and not juveniles affect mouth form? For now we can only speculate, but given that St animals develop slightly faster (Serobyan et al., 2013), there may be a “race” to sexual maturation in emergent populations at low densities. However, as the nematode population increases, there will likely be a commensurate decrease in bacterial populations. When faced with competition from other nematodes, *P. pacificus* has a particular advantage in developing the Eu morph; their expanded dietary range includes other nematode competitors. Indeed, when nematode prey is the only available food source, animals with the Eu morph have longer lifespans and more progeny than animals with the St morph (Serobyan et al., 2014). When resources become depleted as the population size increases, *C. elegans* and other monomorphic nematodes may enter dauer and disperse (Frézal and Félix, 2015). However, in St-biased dimorphic strains of *P. pacificus*, juveniles may switch to the Eu morph in response to adults as a first-level indication of rapidly increasing population size (Figure 6). Then, after prolonged starvation and crowding, worms will presumably enter dauer. By analogy to economic models of population growth (Malthus, 1826, Trewavas, 2002) mouth-form plasticity is a “technological innovation” to temporarily escape a Malthusian resource trap.

The evolution of dimorphic mouth forms is one among myriad nematode ecological strategies. For example, entomopathogenic nematodes release their symbiont bacteria in insect hosts to establish their preferred food source, and the bacteria can release antibiotics to kill off competing bacteria and fungi (Griffin, 2012). Some free-living species, like those of the genus *Oscheius*, may refrain from combat and stealthily feed and reproduce amid warring entomopathogenic species (Campos-Herrera, 2015a). Interspecific killing also occurs in gonochoristic species, in which both mated and virgin males are killed, implying fighting not just for mates but for resources as well (O'Callaghan et al., 2014, Zenner et al., 2014). Different reproductive strategies also exist, and hermaphroditic species have an advantage over gonochoristic species when colonizing a new niche, such as an insect carcass (Campos-Herrera, 2015b). Meanwhile, insect hosts and colonizing nematodes have their own distinct pheromone-based attraction and toxicity (Cinkornpumin et al., 2014; Renahan and Hong, 2017). Finally, the renaissance of *C. elegans* sampling from around the world (Cook et al., 2017, Evans et al., 2016, Félix et al., 2013, Petersen et al., 2014, Poullet and Braendle, 2015) is rapidly building a resource of wild isolates that will almost certainly have different and fascinating ecologies. We hope our method for labeling and then combining different nematode populations on the same plate will aid in studies to identify these strategies. Perhaps the time is also ripe to complement these studies with more sophisticated ecological modeling that can lead to testable hypotheses.

Although beyond the scope of this manuscript, the cross-generational communication we observed could in principle reflect an intended signal from adults to juveniles, i.e., kin selection (Bourke, 2014). However, we favor a more simplistic view that juveniles have evolved to recognize late-stage metabolites. Regardless of these interpretations, our results argue that age classes are a critical factor in density-dependent plasticity, as has been theorized in density-dependent selection (Charlesworth, 1994).

### Limitations of the Study

Given the ubiquity of certain traits in reproductive adults and their contribution to population growth, we suspect similar results will be found in other systems. However, it may depend on the phenotype and system being studied. For example, the population dynamics of this nematode (fast hermaphroditic reproduction) may be sufficiently different from other species such that our findings have limited generalizability. In addition, our method of staining different populations, although fast and easy, is particular to nematodes. Finally, to what extent our ecological interpretations exist in nature remains to be determined.

## Methods

All methods can be found in the accompanying Transparent Methods supplemental file.

## Supplemental Information

Document S1. Transparent Methods, Figures S1–S6, and Tables S1–S4
