# Supplementary material for: Adult Influence on Juvenile Phenotypes by Stage-Specific Pheromone Production
Source: iScience. 2018 Nov 20;10:123–34. doi: 10.1016/j.isci.2018.11.027 (PMC6279967; doi:10.1016/j.isci.2018.11.027)
Supplement: Document S1. Transparent Methods, Figures S1–S6, and Tables S1–S4 [file mmc1.pdf]

ISCI, Volume *10*

Supplemental Information

Adult Influence on Juvenile Phenotypes by Stage-Specific Pheromone Production

Michael S. Werner, Marc H. Claaßen, Tess Renahan, Mohannad Dardiry, and Ralf J. Sommer

Supplemental Information

Transparent Methods

Nematode strains and husbandry

P. pacificus Wild-type RS2333 (California) and RSC017 (La Réunion) strains were kept on 6 cm nematode growth media (NGM) plates seeded with OP50 and kept at 20°C. RSC017 is highly St and does not predate on other nematodes, and thus was used for biological assays instead of the highly Eu, predatory RS2333. To induce dauer, mixed-stage plates with little to no OP50 were washed with M9 and the resulting worm pellets were used in a modified 'White Trap' method. Worm pellets were placed on killed *Tenebrio molitor* grubs and dispersing dauers were collected in surrounding MilliQ water. Age of dauers ranged from one week to one month.

Dye staining

A stock solution of Neutral Red was prepared by dissolving 0.5 mg in 10 ml 5% acetic acid and stored at -20°C. Working solutions were prepared by 100x dilution in M9, aliquoted, stored at -20°C, and thawed directly before use. Working solutions were kept for approximately 1 month. Stock solutions of 10 mM CellTracker Green BODIPY were made in DMSO and stored at -20°C. J2s were prepared from 20-40 x 6 cm plates 6 days after passaging 5 worms to each plate on 300 µl OP50. Worms were washed from plates with M9 into a conical tube, and then filtered through 2 x 20 µM filters (Millipore) placed between rubber gaskets. The flow-through contained mostly J2 and some J3, which were pelleted by centrifugation, 8 seconds on a table-top eppendorf centrifuge 5424, reaching approximately 10,000 x g. The older/larger adult worms

remained on the filters, and were washed into a 50 ml conical tube with ~2 ml M9. Adults were then isolated by transferring worms to a 15 ml conical, and allowing them to swim/sink to the bottom of the tube. Adults reach the bottom faster than younger stages do, and after 3-5 rounds of removing supernatant and re-suspending in 2-3 ml M9, the pellet contains almost exclusively adults, which were re-suspended in 1 ml M9/50 µM Green BODIPY (Thermo Fisher). The J2 pellet was either directly re-suspended in 1 ml Neutral Red working solution, or in 1 ml M9 and split to two tubes, re-centrifuged, and re-suspended in 1 ml working solution Neutral Red (0.005% in M9) or 1 ml M9/50 µM Green BODIPY (Thermo Fisher). For the intermediate time point juveniles (J3s and some J4s), J2s isolated from 20 µM filtering were placed back on agar plates containing 300 µl OP50 bacterial food and grown for another 24 hours, and then washed from plates in M9 and re-filtered through 5 µM filters, then re-suspended in 1 ml 50 µM Green BODIPY (Thermo Fisher). Each tube was rotated for 3 hours in the dark at 20°C, then washed by centrifugation as before, and re-suspended in 1 ml M9. This was repeated 3-4x until the dye was no longer visible in the worm pellet. Then, the concentration of worms per microliter was determined by aliquoting 2 µl onto a glass coverslip in 5 technical replicates, and counted under a dissecting microscope. Finally the appropriate number of animals was added to 6 cm plates that had been previously seeded with 300 µl OP50, and incubated at 20°C. After 3 days, 100% of worms exhibited Neutral Red staining (*n*=50, Supplementary Figure 3). Dauers and J2s recovered after Neutral Red staining developed at the same developmental speed (3-4 days) and with the same mouth-form ratio as control worms recovered side-by-side (100% St for both, Supplementary Figure 4, *n*=30). Dauers and J2s stained with CellTracker Green BODIPY (50 µM) (Thermo) were similar, although less efficiently stained compared to Neutral Red. On day 4, 90% retained intestinal fluorescence (Supplementary Figure 3), and brightness decreased with the number of days. J2s in +/- 50 µM CellTracker Green BODIPY also developed at equivalent rates and mouth-form ratios (Supplementary Figure 4). Lower than 25 µM did not yield strongly

fluorescent worms after three hours. CellTracker Blue CMAC (Thermo Fisher) was also used at 50 µM and imaged 3 days post-staining for *P. pacificus*, and one day post-staining for *C. elegans*. However, due to the higher fluorescent background in the blue light spectrum in both *P. pacificus* and *C. elegans*, we performed all experiments using only Neutral Red and CellTracker Green BODIPY.

Microscopy

All images were taken on a Zeiss Axio Imager 2 with an Axiocam 506 mono, and processed using Zen2 pro software. Image brightness and contrast were enhanced in ImageJ with a minimum displayed value of 10 and maximum of 100 for all images in Figure 2, and Supplementary Figures 1 and 2, and a minimum of 21 and maximum of 117 for Supplementary Figure 3. The following exposure times were used for all images: Cy3 (peak emission = 561, exposure = 80 ms), FITC (peak emission = 519, exposure = 150 ms), Dapi (peak emission = 465, exposure = 80 ms), DIC (exposure = 80-140 ms).

Mixed culture experiments and statistical analysis

We performed the mixed culture experiments presented in Figure 3 with a minimum total number of counts $n > 100$, from three to five independent biological replicates for J2/24 hr, dauer, and adult competitor experiments, and two for the intermediate (J3/4) juvenile experiment (median counts per replicate for J2/24 hr=29, dauers=27, and adults=21, and avg. J3/4 counts was 75). J2 or dauers were stained with Neutral Red, then added to green-stained J2, dauer, J3/4, or adult populations as described in the 'Dye Staining' method section, on 6 cm plates with 300 µl OP50 and incubated at 20°C. To ensure consistent bacterial food supply, we added 1 ml more overnight OP50-LB to each plate on the following day, then air-dried under a chemical fume hood for 1 hour, then returned the plates to 20°C. On days three to four, we

phenotyped ‘red’ adults that exhibited no ‘green’ staining. To assess whether the age of the ‘green’ surrounding population affects the mouth form of the dependent variable ‘red’ J2s we performed a binomial regression on Eu counts (i.e. “successes”) weighted by the number of counts per replicate, and the stage (juveniles vs. adults) and number added as a fixed effects, using a generalized linear model from the standard statistical package in R:

glm(formula=cbind(Eu,total)~’stage_added’ * ‘#_added’, data=’J2/Da’, family="binomial"))

See Supplementary Table S1 for a table containing the resulting *p* values. The AIC for our models (85.52 for juveniles and 72.32 for dauers) was substantially lower than the null hypothesis (220.16 for J2s and 147.29 for dauers), arguing a reasonable fit. For pair-wise comparisons of the effect of age for a given number of added animals, we performed a post-hoc Fisher's exact test on a contingency table containing the summed counts (n>100) of Eu and St observations against control plates (no added crowding animals). For display, we converted Eu counts into percent of total in Figure 3, with the *p* values for the number of animals added indicated over the relevant column (Significance codes: 0 ‘***’ 0.001, ‘**’ 0.01, ‘*’ 0.05).

Measuring the effect of food depletion on mouth form

To verify that starvation was not a factor in our mixed culture experiments, we added increasing number of J2s to standard 6 cm plates with 300 µl OP50 to rapidly consume bacterial food, and measured both the amount of animals that reached adulthood, and the percent Eu in each population for two biological replicates. To assess the affects of added J2s to each dependent variable we performed a binomial regression with count data weighted by the total number of counts for each replicate:

glm(formula = cbind(reached_adult, total)~thousand_J2s, data=data_2, family="binomial"))

p values indicate a significant difference in percent reaching adult as a function of J2s added, but not in percent Eu (Table S1 bottom frame).

Supernatant collection and assays

Strains RS2333, RSC017, and RS2333-*daf-22.1;22.2* were raised in 10 ml liquid culture as in the time-resolved NDMM collections (see below). For each time point, 9 ml of the supernatant was lyophilized overnight, extracted again overnight with 90% ethanol (diluted in Millipore water) while being stirred, and centrifuged (4000 x g, 10 min, 4°C). The solvent was evaporated and the solid re-dissolved with 1 ml Millipore water. This clear extract was then directly used for the assays. One ml of the supernatant was cleaned for HPLC-MS analysis for quality control, as described in HPLC-MS sample preparation below. For the assays, RSC017 was synchronized by bleaching (Werner et al., 2017) and added to plates seeded with 300 µl OP50. The supernatants were added to the RSC017 J2s in two 500 µl increments (for a total of 1 ml supernatant) and dried for 30 minutes in a sterile hood after each addition. Plates were kept at 20°C and adult mouth forms were screened three days later. To determine significance a Fisher Exact test was performed on summed count data relative to S-medium control contingency tables, and the data are presented for representation as percentages in Figure 4.

HPLC-MS sample preparation for exo-metabolome and time resolved analysis

To collect staged pheromone profiles, we seeded 35 x 6 cm plates with 5 worms each, and bleached 5-6 days later when gravid to collect eggs/J1s. These were then added to 6 x 10 ml flasks with OP50 as described in Werner et al., 2017 (Werner et al., 2017). Then at 24, 48, or 72 hr time intervals, supernatants were obtained by centrifugation (>4,000 x g, 4°C for 10 minutes). 1 ml supernatant was adsorbed onto a SPE-C8 cartridge (Thermo Scientific Hypersep C8 100 mg/1ml), conditioned with 1 ml MeOH followed by 2 ml Millipore water. The adsorbed material was then washed with 200 µl water and subsequently eluted with 200 µl MeOH. This extract was then measured directly via HPLC-qTof MS (Bruker ImpactII).

HPLC-MS measurement

20 µl extract was injected into a Thermo UltiMate 3000 HPLC equipped with a Sigma-Aldrich Ascentis Express C18 2.7 µm 10 mm x 4.6 mm column at 20°C with a flow of 500 µl/min. All MS measurements have been performed in negative ion mode and molecules are detected as $[M-H]^-$ Ions. The solvent gradient started with 5% acetonitrile (ACN)/ 95% water (both containing 0.1% formic acid) for 2 minutes. After this equilibration step, the ACN proportion was increased to 65% over 8 min, then to 100% ACN in 1.2 minutes followed by a hold step for 8.8 minutes. Afterwards, the system was flushed to 5% ACN with 2 minutes equilibration for a total of 22 minutes. For calibration, a sodium formate cluster building solution was automatically injected in the first 2 minutes of each run. Data analysis was performed with TASQ version 1.0 from Bruker Daltonics. Extracted ion chromatograms for each well-known compound with a mass width of 0.1 m/z and time slices of 0.5 minutes around the expected retention time were produced after calibrating and baseline correction. Assignment errors were corrected with the provided MRSQ value, and areas under the curve were calculated from the integral of each peak.

Statistical analysis of NDMMs

NDMM levels were compared simultaneously against strains and developmental stages by a linear model in R: lm(‘NDMM’ ~ ‘developmental stage’ * ‘strain’, data=’data.frame’)). In essence, the linear model regressed the abundance of NDMMs against stage and strain as fixed effects. *P* values between stages and strains were adjusted for multiple testing by a false discovery rate correction (FDR). The level of fit between linear vs. exponential growth was determined by the Akaike information criterion (AIC). The lowest AIC for iterations of different exponents (n=1,2,3…) was used for comparison to the simple linear model. While significant in both cases, for consistency we present the original *p* values from the original linear model in Table S2.

Supplemental Figure Legends

Figure S1, related to Figure 2. Vital dye staining of *Pristionchus pacificus*.

(A) Control *P. pacificus* imaged with Cy3, FITC, and DAPI filters, and a merge with Differential Interference Contrast (DIC). Histogram on the right represents quantification of intensity with each filter. (B) Same as (A) but stained with 0.005% Neutral Red, (C), 50 µM CellTracker Green BODIPY (Thermo Fisher), or (D) 50 µM CellTracker Blue CMAC Dye (Thermo Fisher). J2s were stained (see Transparent Methods), and ensuing adult animals were imaged 3 days later on a Zeiss Axio Imager 2 with an Axiocam 506 mono, and processed using Zen2 pro software. Image brightness and contrast were enhanced in ImageJ for display, with a minimum displayed value of 10 and maximum of 100 for all images. Note that while Neutral Red and CellTracker Green staining are bright and specific to their respective channels, CellTracker Blue is indistinguishable from background fluorescence.

Figure S2, related to Figure 2. Vital dye staining of *Caenorhabditis elegans.*

(A-D) Same as Supplementary Figure 1, but with *C. elegans*.

Figure S3, related to Figure 2. Vital dye staining of *P. pacificus* dauers, and duration of staining. (A) Control *P. pacificus* dauer imaged with DIC, Cy3, and FITC filters. (B) Dauers stained with either 0.005% Neutral Red or 50 µM CellTracker Green BODIPY and imaged immediately after staining with DIC, Cy3, and FITC filters and merged with DIC. Images were taken using Zeiss Axio Imager 2 with an Axiocam 506 mono, processed using Zen2pro software, and adjusted in ImageJ, with a display value minimum of 21 and maximum of 117.

(C-G) 50 µM CellTracker Green BODIPY and 0.005% Neutral Red-stained J2s were imaged every day for five days. Percent of individuals retaining the dyes are shown in panels next to each microscope image for each day. Both stains are seen in all organisms for three days; Neutral Red (NR) persists for at least five, while the number of Green BODIPY (GB) –stained worms drops on day four. All images are merged with DIC, n=31 GB, 63 NR day 1, 68 GB, 56 NR day 2, 50 GB, 50 NR day 3, 50 GB, 50 NR day 4, 50 GB, 50 NR day 5.

Figure S4, related to Figure 2. Vital dye staining does not affect *P. pacificus* mouth form or development.

(A) Neutral Red and CellTracker Green BODIPY-stained J2s reach adulthood at the same rate as unstained J2s (3 days). (B) All of the J2s stained retain the dye in adulthood in the intestine. (C) Neither dye affects mouth form; both unstained and stained worms remain 100% St (n=30). (D-F) Same as for (A-C) except with dauers instead of J2s, and only with Neutral Red. (G) Developmental rate of J2 unstained, Neutral Red-stained (NR), and CellTracker Green BODIPY-stained (GB) RSC017 every 12 hours post-J2 staining. Two biological replicates, n=60. To see if there were significant differences between stained and un-stained, a Fisher's Exact test was performed on summed counts of each stage (all p>0.05) (H) Staging of RSC017 worms from liquid culture at the relevant time points, 24 hrs, 48 hrs, and 72 hrs. Error bars represent standard error of the mean for 3 biological replicates, n>100 animals counted per replicate.

Table S1, related to Figure 3. Table of binomial regression *p* values for crowding assays.

Significance p values from binomial regression of vital-dye method for age and number added, and from binomial regression of number-reaching-adult and Eu counts, for each number of individuals added relative to 1,000 individuals added (see Transparent Methods for details).

Figure S5, related to Figure 5. Pheromone profiling quality control.

(A) Extracted ion traces (width 0.1 m/z) of 11 of the 12 NDMMs used in this publication from a seven-day mixed-stage sample, double peak of 247.12 m/z indicate isomeric structures (Part#9/Ascr#9). (B) Example of an averaged spectrum over a calibration segment; sodium-formate cluster building solution was used to ensure high mass accuracy in each run. (C) Comparison of an endometabolome sample from a seven- day mixed-stage cultured compared to the endometabolome of eggs, produced by using bleached eggs from 80 x 60 mm plates.

Table S2, related to Figure 5. Table of linear regression *p* values with FDR corrections for strain and stage comparison of NDMM levels. FDR-corrected and uncorrected *p* values from linear regression of *P. pacificus* NDMMs (alternating grey background between NDMMs for clarity). Red values indicate FDR<0.05.

Table S3, related to Figure 5. P values from pairwise comparison of dasc#1, npar#1, and ascr#9 throughout development. Significance assessed with a two-tailed student's *t*-test. Top table indicates comparison of raw pheromone levels experienced by worms, and the bottom table indicates comparison of volume-normalized pheromone levels (normalized data from WormSizer (Moore et al., 2013), Fig. S6B-D).

Figure S6, related to Figure 5. Enzyme that synthesizes NDMMs is transcriptionally regulated during development, and volume normalization of pheromones. (A) Comparison of *daf-22.1* (FPKM) by RNA-seq through different stages of development, data from Baskaran et al., 2015 (Baskaran et al., 2015). A two-sided students *t*-test was performed between 56-68

hours (J4-adults) and 22 hours (J2s) (Significance codes: 0 '***' 0.001, '**' 0.01, '*' 0.05). (B) Representative images of worms raised in liquid culture at 24 hrs, 48 hrs, and 72 hrs. (C) Comparison of worm volumes (picoLiters) for 24 hrs, 48hrs, and 72 hrs, using WormSizer (Moore et al., 2013). (D) Time-resolved NDMM levels of RSC017 normalized by worm volume (upper graph) and unnormalized (lower graph, also shown in Figure 5B). Data is presented as the mean of nine biological replicates and error bars represent standard error of the mean (SEM). In the upper graph, levels were normalized to worm volume based on the data shown in (C).

Table S4, related to Figure 5. Raw and volume-normalized data of RSC017 pheromones, in absolute value of area under the curve. Normalization of 48 hr and 72 hr time point abundances relative to 24 hrs. Average volumes obtained by WormSizer (Moore et al., 2013)(Figure S6B-C).

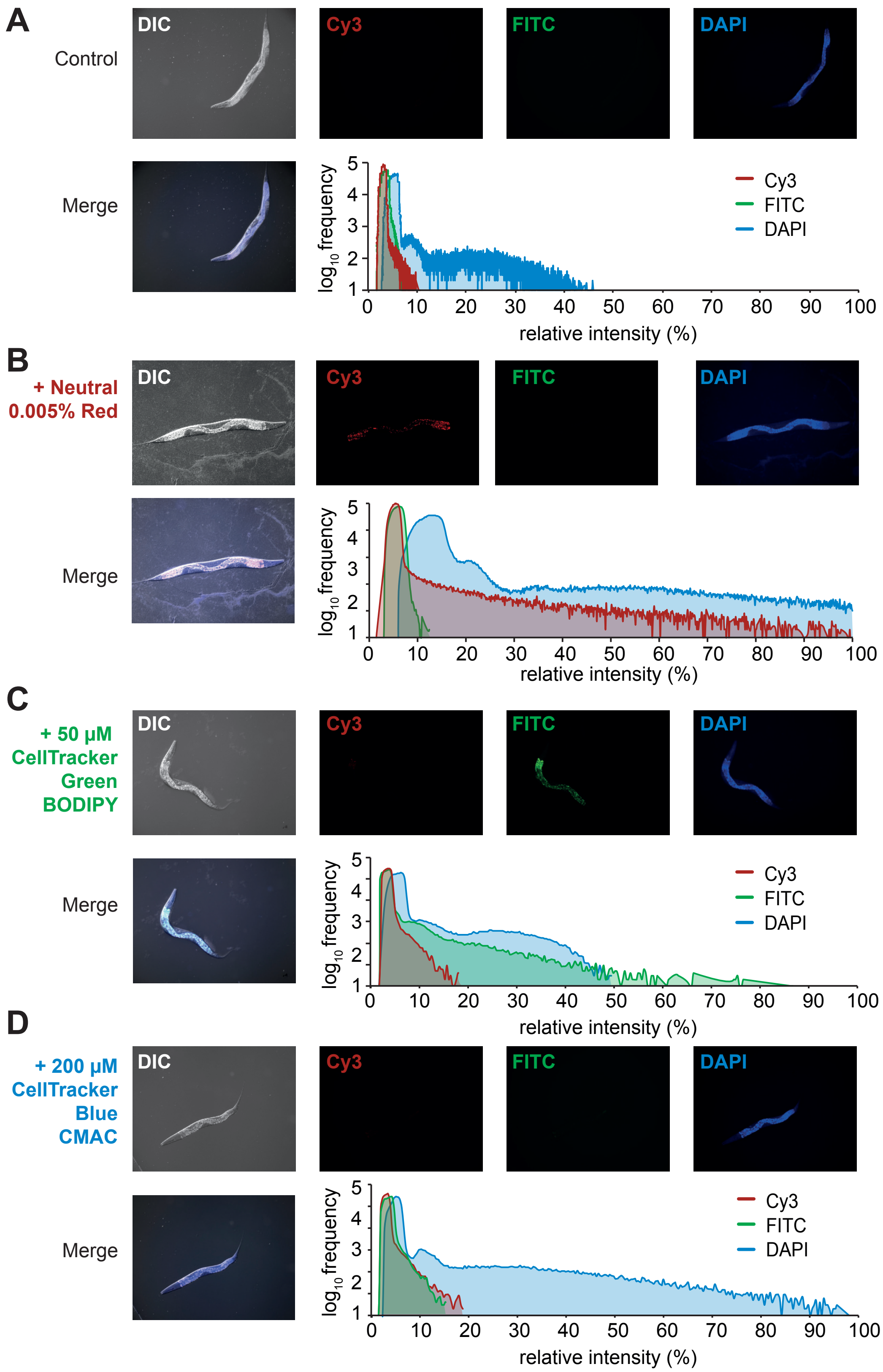

Figure S1, related to Figure 2. Vital dye staining of *Pristionchus pacificus*.

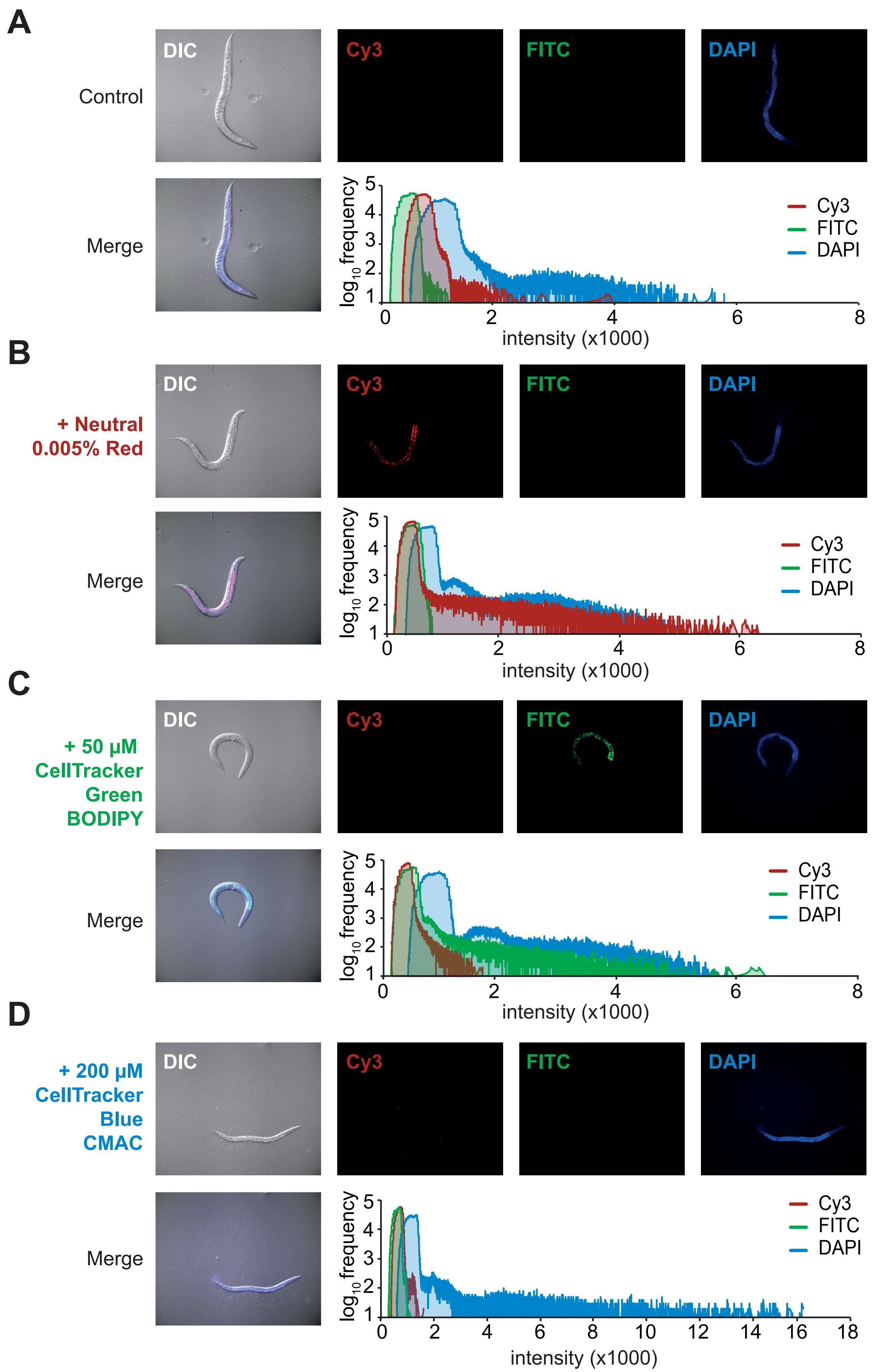

Figure S2, related to Figure 2. Vital dye staining of *Caenorhabditis elegans.*

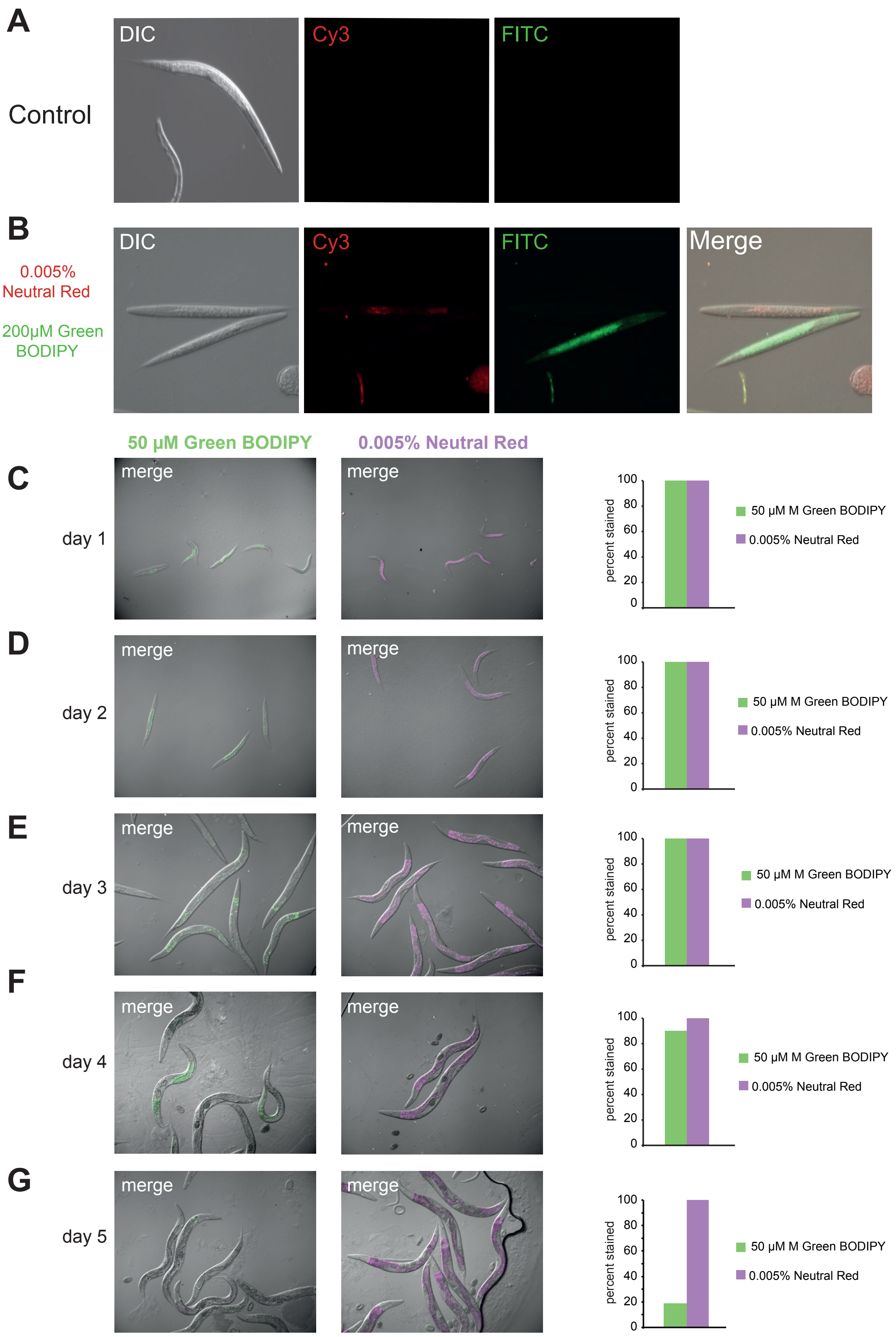

Figure S3, related to Figure 2. Vital dye staining of *Pristionchus pacificus* dauers, and duration of staining.

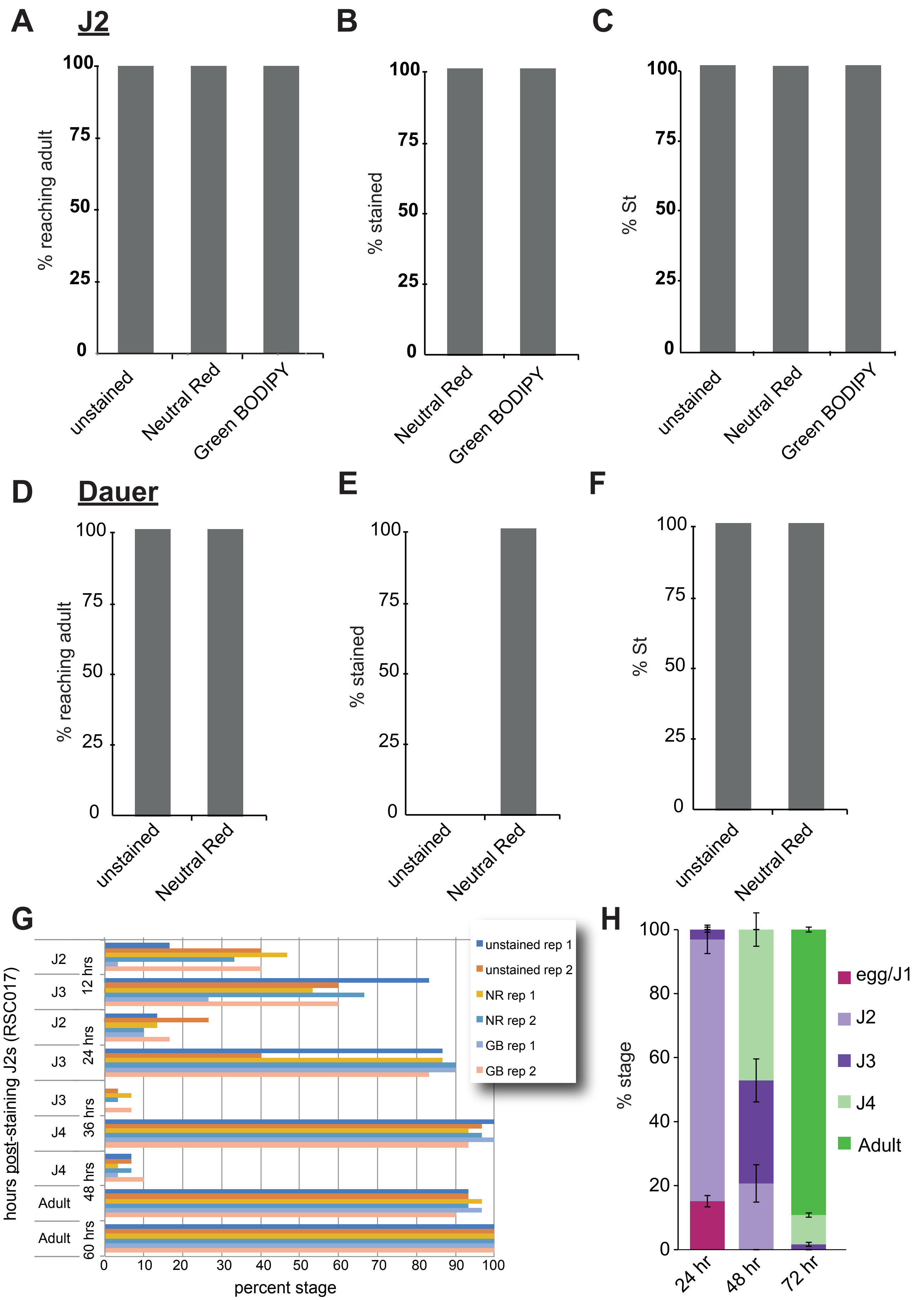

Figure S4, related to Figure 2. Vital dye staining does not affect P. pacificus mouth form or development.

effect of population age on mouth form of developing juveniles

binomial regression	*p* value red-stained J2s	*p* value red-stain dauers
**stage added (adults vs. juveniles)**	0.0132	0.002955
**number added**	4.28e-13	0.000404

effect of number of peers on development and mouth form (proxy for potential starvation effects on mouth form)

binomial regression	*p* value for development (relative to 1,000)	*p* value for Eu (relative to 1,000)
**3,000 J2s added**	0.3408	1.0
**4,000 J2s added**	0.0424	1.0
**5,000 J2s added**	6.06E-14	0.99
**10,000 J2s added**	4.09E-14	0.99

Table S1, related to Figure 3. Table of binomial regression p values for vital-dye method and excess crowding.

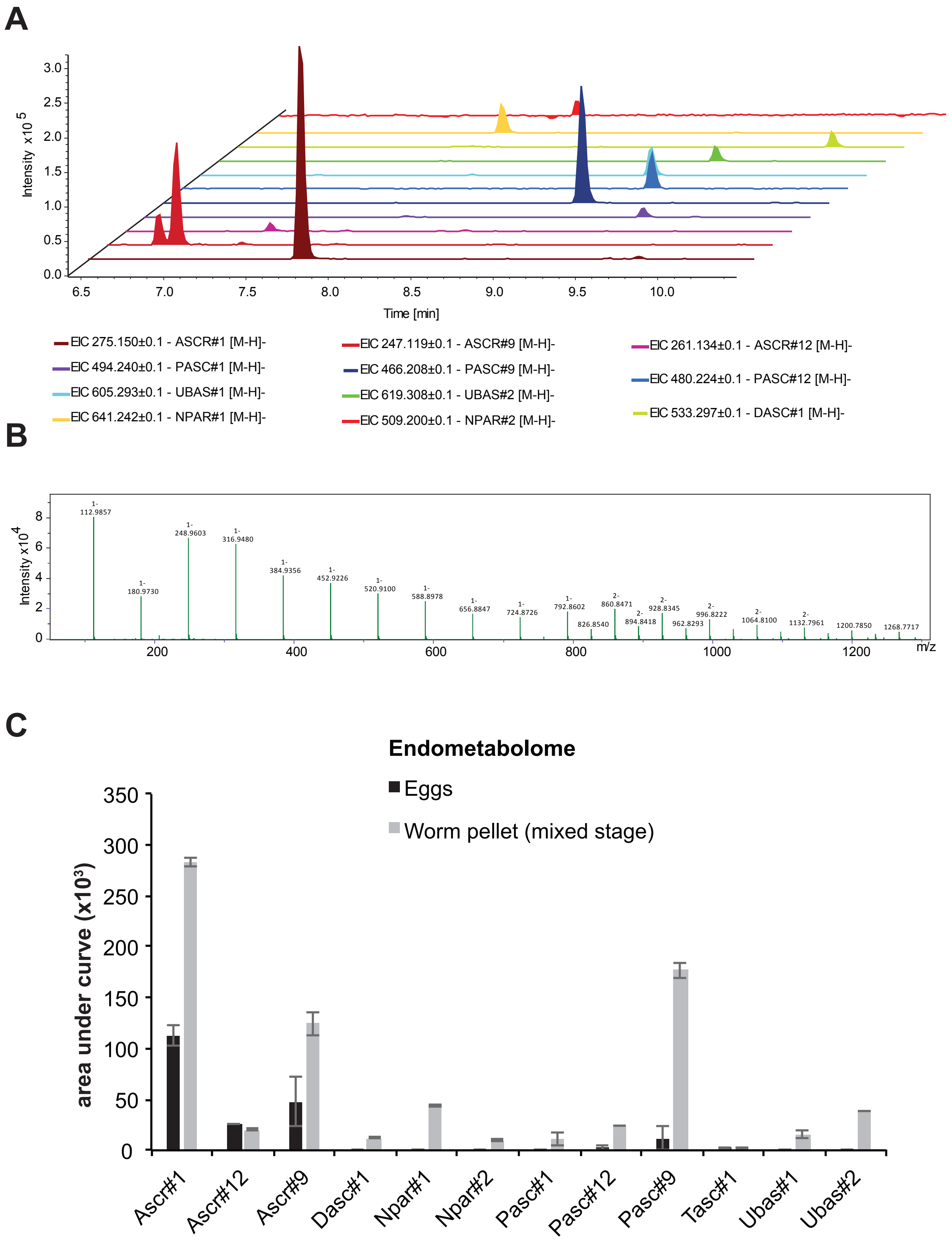

Figure S5, related to Figure 5. Pheromone profiling quality control

NDMM comparison	pvalue	fdr corrected
ascr1_stage	0.4733	0.774490909
ascr1_strain	0.0429	0.110314286
ascr1_stage:strain	0.031	0.085846154
ascr9_stage	3.79E-05	0.0002274
ascr9_strain	0.651	0.778064516
ascr9_stage:strain	0.272	0.50148
ascr12_stage	0.0029	0.01404
ascr12_strain	0.0897	0.201825
ascr12_stage:strain	0.0302	0.085846154
dasc1_stage	9.62E-08	8.66E-07
dasc1_strain	0.11363	0.240628235
dasc1_stage:strain	0.00351	0.01404
npar1_stage	0.0033	0.01404
npar1_strain	0.9426	0.984
npar1_stage:strain	0.6355	0.778064516
npar2_stage	0.0516	0.12384
npar_2strain	0.984	0.984
npar2_stage:strain	0.9716	0.984
pasc1_stage	0.449	0.769714286
pasc1_strain	0.753	0.847125
pasc1_stage:strain	0.564	0.778064516
pasc9_stage	0.616	0.778064516
pasc9_strain	0.267	0.50148
pasc9_stage:strain	0.523	0.778064516
pasc12_stage	0.6122	0.778064516
pasc12_strain	0.2786	0.50148
pasc12_stage:strain	0.67	0.778064516
tasc1_stage	0.522	0.778064516
tasc1_strain	0.862	0.940363636
tasc1_stage:strain	0.57	0.778064516
ubas1_stage	3.13E-12	1.13E-10
ubas1_strain	0.00538	0.019368
ubas1_stage:strain	6.69E-08	8.03E-07
ubas2_stage	1.34E-11	2.41E-10
ubas2_strain	0.00711	0.023269091
ubas2_stage:strain	6.18E-07	4.45E-06

Table S2, related to Figure 5. Table of linear regression *p* values with *FDR* correction for strain and stage comparison of NDMM levels.

RS2333	dasc#1	npar#1	ascr#9
72 hrs compared to 24 hrs	5.75E-07	3.47E-05	1.03E-04
72 hrs compared to 48 hrs	5.71E-03	1.76E-01	1.97E-01
RSC017	dasc#1	npar#1	ascr#9
72 hrs compared to 24 hrs	2.55E-02	3.66E-03	2.03E-02
72 hrs compared to 48 hrs	2.12E-01	3.66E-01	1.04E-01

Volume normalized

RS2333	dasc#1	npar#1	ascr#9
72 hrs compared to 24 hrs	5.75E-07	3.47E-05	1.02E-03
72 hrs compared to 48 hrs	1.44E-02	2.92E-01	6.21E-01
RSC017	dasc#1	npar#1	ascr#9
72 hrs compared to 24 hrs	2.55E-02	3.66E-03	4.34E-02
72 hrs compared to 48 hrs	2.71E-01	5.46E-01	1.70E-01

Table S3, related to Figure 5. P values from pairwise comparison of dasc#1, npar#1, and ascr#9 throughout development.

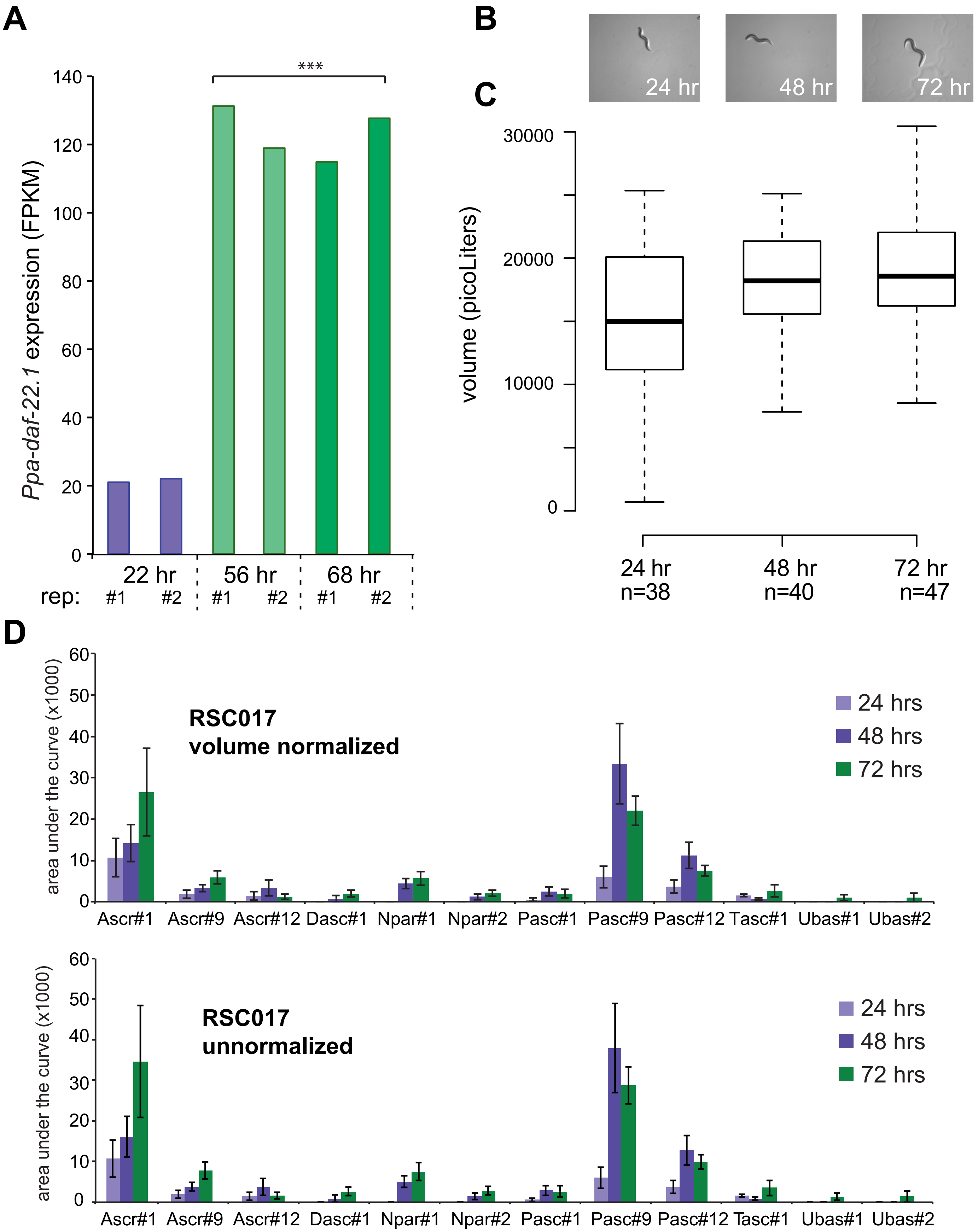

Figure S6, related to Figure 5. Enzyme that synthesize NDMMs is transcriptionally regulated during development, and volume normalization of pheromones.

STAGE	DASC1	NPAR1	Pasc9	Ascr1	Ascr12	Ascr9	Npar2	Pasc1	Pasc12	Tasc1	Ubas1	Ubas2
24	0	0	0	0	0	0	0	0	0	1610	0	0
24	0	0	0	4489	0	0	0	0	0	1214	0	0
24	0	0	0	0	0	0	0	0	0	1769	0	0
24	0	0	0	22265	0	4301	0	0	0	1169.5	0	0
24	0	0	0	28319.5	0	5450	0	0	0	1871.5	0	0
24	0	0	7193.5	35197.5	8299.5	7177	0	0	9476	3918	0	0
24	0	0	16048.5	3929.5	5028.5	0	0	0	8318	969.5	0	0
24	0	0	19293.5	2386.5	0	0	0	1657.5	11094	999	0	0
24	0	0	11623.5	0	0	0	0	3667.5	4799.5	949.5	0	0
48	7800	8901	111298	7866	0	4250	6050	8486	35583.5	2827	0	0
48	0	8393	54479	7660	0	7077	5605	6699	19222.5	1047.5	0	0
48	0	10347	32381.5	11133	0	4339	0	6513	11901.5	2324	0	0
48	0	0	13819	34084	16659.5	5087	0	0	5916	1217	0	0
48	0	0	6893	40108	12167	7298	0	0	0	0	0	0
48	0	0	6766	32972	5415	6235.5	0	0	0	0	0	0
48	0	7635.5	56471.5	6725	0	0	1522	3957	17663.5	400.5	0	0
48	0	7036	29685.5	4781	0	0	0	0	13964.5	0	0	0
48	0	3205.5	29656	0	0	0	0	0	10977	0	0	0
72	0	16111.5	45664.5	9007	0	7593.5	5065	10394.5	17614.5	2243	0	12581
72	6321	9157.5	36161.5	7275	0	5649	5062	8322	13492	562.5	0	0
72	4475.5	17381	51388	7354.5	0	7472	7192	5269.5	12932	1192	0	0
72	7400.5	10075	25671	93903	6060	22877	6485	0	8342	12416	6377.5	0
72	0	0	9248.5	61584	3670.5	7879	0	0	0	14621	0	0
72	5861	0	13904	107297	4907.5	8875	0	0	5734	0	5697.5	0
72	0	0	20159.5	12767.5	0	0	0	0	9426	0	0	0
72	0	7294.5	28800	6249	0	3823	0	0	7802	544	0	0
72	0	7454.5	28094	6695.5	0	6082	1696.5	0	13884.5	201	0	0

RSC017 pheromone levels

STAGE	DASC1	NPAR1	Pasc9	Ascr1	Ascr12	Ascr9	Npar2	Pasc1	Pasc12	Tasc1	Ubas1	Ubas2
24	0	0	0	0	0	0	0	0	0	1610	0	0
24	0	0	0	4489	0	0	0	0	0	1214	0	0
24	0	0	0	0	0	0	0	0	0	1769	0	0
24	0	0	0	22265	0	4301	0	0	0	1169.5	0	0
24	0	0	0	28319.5	0	5450	0	0	0	1871.5	0	0
24	0	0	7193.5	35197.5	8299.5	7177	0	0	9476	3918	0	0
24	0	0	16048.5	3929.5	5028.5	0	0	0	8318	969.5	0	0
24	0	0	19293.5	2386.5	0	0	0	1657.5	11094	999	0	0
24	0	0	11623.5	0	0	0	0	3667.5	4799.5	949.5	0	0
48	6859.790284	7828.076066	97882.17167	6917.834663	0	3737.706244	5320.734771	7463.100045	31294.27533	2486.234248	0	0
48	0	7381.310238	47912.11729	6736.665843	0	6223.940492	4929.374941	5891.504502	16905.42548	921.2346567	0	0
48	0	9099.77565	28478.24347	9791.03144	0	3815.97821	0	5727.924887	10466.89667	2043.86572	0	0
48	0	0	12153.26179	29975.52462	14651.36875	4473.81451	0	0	5202.887092	1070.303176	0	0
48	0	0	6062.119798	35273.39342	10700.39338	6418.301217	0	0	0	0	0	0
48	0	0	5950.428341	28997.56477	4762.277486	5483.874656	0	0	0	0	0	0
48	0	6715.119066	49664.44193	5914.370469	0	0	1338.538566	3480.024379	15534.34688	352.2238473	0	0
48	0	6187.88262	26107.21852	4204.69966	0	0	0	0	12281.22326	0	0	0
48	0	2819.109969	26081.27444	0	0	0	0	0	9653.835634	0	0	0
72	0	12340.85154	34977.427	6899.050356	0	5816.358263	3879.61475	7961.827348	13492.09753	1718.060392	0	9636.610695
72	4841.667292	7014.328149	27698.45781	5572.398284	0	4326.938544	3877.316854	6374.364057	10334.40517	430.8555374	0	0
72	3428.078147	13313.24461	39361.42997	5633.292533	0	5723.293467	5508.823155	4036.254674	9905.46455	913.0307566	0	0
72	5668.526941	7717.101403	19663.09778	71926.44894	4641.750323	17522.99045	4967.285618	0	6389.683365	9510.226404	4884.944337	0
72	0	0	7084.031	47171.21318	2811.476	6035.041385	0	0	0	11199.18011	0	0
72	4489.323208	0	10649.98292	82185.7895	3758.9752	6797.942923	0	0	4392.045603	0	4364.087865	0
72	0	0	15441.47948	9779.463242	0	0	0	0	7219.989859	0	0	0
72	0	5587.334609	22059.80351	4786.517783	0	2928.285723	0	0	5976.062049	416.6851775	0	0
72	0	5709.889073	21519.03194	5128.521334	0	4658.601562	1299.460301	0	10635.04659	153.9590454	0	0

RSC017 volume normalized pheromone levels

Table S4, related to Figure 5. Raw and normalized data of RSC017 pheromones, in absolute value of area under the curve.
